# A novel FAM83G variant from palmoplantar keratoderma patient disrupts WNT signalling via loss of FAM83G-CK1α interaction

**DOI:** 10.1098/rsob.240075

**Published:** 2024-07-24

**Authors:** Lorraine Glennie, Marta Codina Solà, Mar Xunclà, Gloria Aparicio Español, Elena Garcia-Arumí, Eduardo Fidel Tizzano, Nicola T. Wood, Thomas J. Macartney, Amaia Lasa-Aranzasti, Gopal P. Sapkota

**Affiliations:** ^1^ Medical Research Council Protein Phosphorylation & Ubiquitylation Unit, School of Life Sciences, University of Dundee, Dundee DD1 5EH, UK; ^2^ Department of Clinical and Molecular Genetics, Vall d’Hebron Barcelona Hospital Campus, Barcelona, Spain; ^3^ Medicine Genetics Group, Vall d’Hebron Institut de Recerca (VHIR), Vall d’Hebron Hospital Universitari, Barcelona, Spain; ^4^ Servicio Dermatología, Vall d’Hebron Hospital Universitari, Barcelona, Spain

**Keywords:** FAM83, palmoplantar keratoderma, hyperkeratosis, CK1, WNT, FAM83G

## Abstract

Palmoplantar keratoderma (PPK) is a multi-faceted skin disorder characterized by the thickening of the epidermis and abrasions on the palms and soles of the feet. Among the genetic causes, biallelic pathogenic variants in the *FAM83G* gene have been associated with PPK in dogs and humans. Here, a novel homozygous variant (c.794G>C, p.Arg265Pro) in the *FAM83G* gene, identified by whole exome sequencing in a 60-year-old female patient with PPK, is reported. The patient exhibited alterations in the skin of both hands and feet, dystrophic nails, thin, curly and sparse hair, long upper eyelid eyelashes, and poor dental enamel. FAM83G activates WNT signalling through association with ser/thr protein kinase CK1α. When expressed in FAM83G^−/−^ DLD1 colorectal cancer cells, the FAM83G^R265P^ variant displayed poor stability, a loss of interaction with CK1α and attenuated WNT signalling response. These defects persisted in skin fibroblast cells derived from the patient. Our findings imply that the loss of FAM83G-CK1α interaction and subsequent attenuation of WNT signalling underlie the pathogenesis of PPK caused by the FAM83G^R265P^ variant.

## 1. Introduction

Palmoplantar keratoderma (PPK) is a heterogeneous skin disorder [1]. Patients commonly display thickening of the skin on the palms and soles of the feet, which can be accompanied by other symptoms. Severe PPK is also associated with Olmsted syndrome, where patients have digit deformities on account of extremely thickened skin, which can lead to autoamputation [2]. Hereditary PPK can be identified at infancy or in young adults as diffuse, focal or striated patterns of hardened thick skin that is often reported to be painful [3]. Variants in keratin genes have been identified in PPK patients, including *KRT1,* which encodes keratin-1 in the differentiating suprabasal epidermis at the outer layer of the skin [4], and *KRT16*, which encodes keratin-16 in proliferating keratinocytes [5–7]. Several variants in the cadherin-type protein desmoglein 1 (DSG1), which is abundant in the upper layer of epidermis, have also been reported in PPK patients [8]. *FAM83G* missense variants were reported in two siblings [9] and a breed of dogs [10] with PPK. The two siblings, carrying *c.101C>A, p.(Ala34Glu),* missense mutation leading to the expression of FAM83G^A34E^ protein, presented with thickened and damaged skin in the palms and soles of their feet and exuberant hair [9]. Similarly, a missense variant in *FAM83G* leading to the expression of FAM83G^R52P^ protein in dogs led to a diagnosis of hereditary footpad hyperkeratosis [10], with the reported phenotypes similar to human patients with PPK [9]. Interestingly, transgenic mice that had a substantial deletion of the *FAM83G* gene locus displayed spontaneous woolly hair phenotype reminiscent of PPK patients and dogs [11]. The exact molecular causes of hereditary and sporadic PPK remain to be fully elucidated.

FAM83G belongs to the FAM83 family of poorly characterized proteins that anchor isoforms of the CK1 family of ser/thr kinases to specific subcellular compartments through the conserved DUF1669 domain [12,13]. The CK1 family of kinases regulates a plethora of cellular processes, including circadian rhythms, WNT signalling and cell division cycle [14]. It has been shown that FAM83F–CK1α and FAM83G–CK1α interaction is critical for the activation of canonical WNT signalling [15,16], and FAM83D–CK1α interaction at the mitotic spindle is essential to ensure proper spindle positioning and timely cell division [17]. The two reported PPK mutations, FAM83G^A34E^ and FAM83G^R52P^ in humans and dogs, respectively, were both previously shown to abolish the interaction with CK1α and led to attenuation of WNT signalling in *Xenopus* embryos and human cell lines [18]. Here, a novel *FAM83G* homozygous variant (c.794G>C) was identified from a 60-year-old female patient diagnosed with PPK which causes Arg265 to Pro substitution (p.Arg265Pro) on the FAM83G protein. Detailed medical indications of the patient are presented, including images of the affected areas. A thorough molecular and functional characterization of the FAM83G^R265P^ variant was also performed in DLD1 colorectal cancer cells as well as skin fibroblasts derived from the patient. The findings show that the loss of CK1α binding and subsequent inhibition of WNT signalling appear to underlie the pathogenesis of PPK caused by the FAM83G^R265P^ mutation in this patient. Not only does this widen our understanding of how FAM83G–CK1α complexes function in WNT signalling but reinforces the significance of dysregulated WNT signalling to the development of PPK.

## 2. Material and methods

### 2.1. DNA sequencing analyses

Given the patient’s clinical presentation (PPK), she was referred from the dermatology unit of Vall d’Hebron Hospital to the Clinical Genetics Unit of Vall d’Hebron Hospital to consider a genetic study. Therefore, the inclusion criteria for the patient’s whole-exome study were as follows: a patient with suspected genetic disease (PPK) and multiple known genes associated with the pathology. DNA was extracted from peripheral blood cells derived from the proband (affected patient), both parents and two unaffected sisters following written informed consent, which was approved by the Vall d’Hebron University Hospital ethics committee. Single exome sequencing was performed in the proband using the xGen Exome Panel v2.0 (IDT). Sequencing was performed on an Illumina Novaseq6000 with 100 bp paired-end reads. Reads were aligned to the GRCh37 reference genome using Burrows–Wheeler aligner (version 0.7.3); duplicate reads were marked using Picard MarkDuplicates (version 2.20.8) and aligned reads were then processed using GATK BaseRecalibrator (Genome Analysis Toolkit; version 3.8) to recalibrate base quality scores, according to GATK Best Practices recommendations. Variants were filtered with a minor allele frequency of <0.005 based on gnomAD (Genome Aggregation Database, version 2.0) and prioritized based on the expected model of inheritance (autosomal recessive or *de novo* following autosomal dominant inheritance) and described gene-disease associations, using HPO terms and literature review of candidate genes. All prioritized variants were validated in the proband and segregated on her two unaffected sisters by Sanger sequencing.

### 2.2. Plasmids

All plasmids used in this study are listed below (See table 1).

**Table 1.** All plasmids used in this study.

recombinant DNA	source	identifier
FAM83G–GFP	MRC PPU Reagents and Services	DU29088
FAM83G^R265P^–GFP	MRC PPU Reagents and Services	DU71791
FAM83G	MRC PPU Reagents and Services	DU33460
FAM83G^F296A^	MRC PPU Reagents and Services	DU28044
SV−40 large T-antigen	MRC PPU Reagents and Services	DU40867
pCMV5-gag-pol	Cell Biolabs	RV−111
pCMV5-VSV-G	Cell Biolabs	RV−110

### 2.3. Generation of skin fibroblasts from patients

Skin-punch biopsy was obtained after written informed consent, which was approved by the Vall d’Hebron University Hospital ethics committee, from the PPK patient (FIB04) as well as from two sex- and age-matched individuals with no PPK diagnosis (FIB03 and FIB15). Primary skin fibroblast cultures were established from the PPK patient and controls. Fibroblasts were cultured in Eagle’s minimal essential medium (MEM, Thermofisher Scientific) supplemented with 20% fetal bovine serum (FBS, Gibco), 1% HEPES buffer 1 M (Cytiva), 1% non-essential amino acids (Corning) and 1% penicillin/streptomycin (Gibco). Cultures were established in T25 flasks and then split in T75 and maintained at 37°C in a 5% CO_2_ atmosphere until passage number 4. Cell dissociation for passaging was performed with trypsin–EDTA (Sigma) treatment when cultures reached >95% confluency.

### 2.4. Cell culture

Wild-type (WT) U2OS (HTB−96; ATCC), WT DLD1 (CCL−221; ATCC), FAM83G^−/−^ DLD1 [19] and HEK293FT cells (Invitrogen, R70007) were cultured in Dulbecco’s modified Eagle medium (DMEM) supplemented with 10% (v/v) FBS (Thermo Fisher Scientific), 2 mM l-glutamine (Lonza), 100 U ml^−1^ penicillin (Lonza) and 0.1 mg ml^−1^ streptomycin (Lonza). FIB15, FIB03 and FIB04 fibroblasts were cultured in DMEM supplemented with 20% FBS, l-glutamine, penicillin, and streptomycin. All cells were cultured with 5% CO_2_ in a humidified incubator at 37°C. For passaging, cells were incubated with trypsin/EDTA to detach cells. Cycloheximide (100 µg ml^−1^; Sigma) or GSK3 inhibitor CHIR99021 (5 µM, 6 h; Sigma) was added directly to the cell culture medium.

### 2.5. Generation of stable cell lines by retroviral transduction

pBabeD puromycin vectors (6 μg) were co-transfected with VSV-G (2.8 μg) and gag/pol (3.2 μg) plasmids into a 10 cm diameter dish of ~70% confluent HEK293FT cells. Plasmids were mixed with 24 μL PEI (1 mg ml^−1^) in 1 ml Opti-MEM and incubated at room temperature for 20 min before the mixture was added dropwise to HEK293FT cells. After 24 h of plasmid transfection, fresh DMEM was added to the cells. After 48 h of transfection, viral media were collected using a syringe and filtered through a 0.45 µm filter into a falcon tube. Target cells (70% confluent) were transduced with retroviruses diluted in fresh medium (1 : 2) and polybrene (8 µg ml^−1^) for 24 h. The transduction medium was then replaced with fresh medium supplemented with puromycin (2 µg ml^−1^) for selection of transduced cells.

### 2.6. Immortalization of primary fibroblasts using SV-40 T-antigen expression

Retroviruses encoding SV-40 T-antigen were collected, passed through a 0.45 µm filter and added to target primary fibroblasts (p5) for 24 h as described above, except the selection was carried out with hygromycin (200 µg ml^−1^) until untransduced control cells died. Surviving cells were cultured in DMEM+20% FBS and taken forward as immortalized fibroblast cell stocks.

### 2.7. Preparation of L- and Wnt3A-conditioned medium

Mouse fibroblast L-cells and L-cells stably overexpressing Wnt3A were cultured for three days in 15 cm dishes and medium collected for control-conditioned medium (L-CM) and Wnt3A-conditioned medium (Wnt3A-CM), respectively. The conditioned media were filtered (0.45 µm) prior to stimulation of target cells.

### 2.8. Cell lysis

Cells were harvested by washing twice with ice-cold phosphate-buffered saline (PBS) before scraping cells into ice-cold NP-40 cell lysis buffer (50 mM Tris–HCl pH 7.5, 0.27 M sucrose, 150 mM NaCl, 1 mM EGTA, 1 mM EDTA, 1 mM sodium orthovanadate, 10 mM sodium β-glycerophosphate, 50 mM sodium fluoride, 5 mM sodium pyrophosphate and 1% NP-40) supplemented with 1 × cOmplete protease inhibitor cocktail (Roche) and 1 mM DTT. Cell lysates were incubated on ice for 10 min and then clarified by centrifugation at 14 000 rpm for 20 min at 4°C. Protein concentration was measured by Bradford assay and samples were normalized using lysis buffer for further use.

### 2.9. Immunoblotting

Extracts (15–20 µg protein) were resolved by sodium dodecyl sulfate-polyacrylamide gel electrophoresis (SDS-PAGE) and transferred to the polyvinylidene fluoride (PVDF) membrane. Membranes were blocked in TBS-T (50 mM Tris–HCl pH7.5, 150 mM NaCl, 0.2% Tween-20) with 5% (w/v) non-fat milk (Marvel). Membranes were then incubated overnight with agitation at 4°C in 5% (w/v) BSA or non-fat milk/TBS-T with the appropriate primary antibodies at the indicated dilutions provided in Section 2.10. Membranes were then washed for 3 × 10 min with 1×TBS-T before incubation with a secondary antibody for 1 h at room temperature. After 3 × 10 min washes with 1×TBS-T, protein signal was detected using ECL (Merck) and ChemiDoc MP System (Bio-Rad). Quantification of protein signal was performed using Fiji 1.53q (ImageJ), and data were processed using Microsoft Excel before representative graphs were generated from numerical data using GraphPad Prism 9.

### 2.10. Antibodies

Details of all antibodies used in this study are listed below shown below (See table 2).

**Table 2.** Details of all antibodies used in this study.

antibody	source	identifier	dilution
sheep polyclonal anti-GFP	MRC PPU Reagents and Services	S268B	1:1000
rabbit polyclonal anti-FAM83G	Abcam	ab121750	1:1000
sheep polyclonal anti-FAM83F	MRC PPU Reagents and Services	SA103	1:1000
sheep polyclonal anti-CK1α	MRC PPU Reagents and Services	SA527	1:1000
rabbit monoclonal anti-c-myc	Cell Signalling Technology	cat. no. 5605S	1:1000
rabbit polyclonal anti-LRP6^pS1490^	Cell Signalling Technology	cat. no. 2568S	1:1000
rabbit monoclonal anti-LRP6	Cell Signalling Technology	cat. no. 3395S	1:1000
mouse monoclonal active β-catenin (clone 8E7)	Sigma-Aldrich	cat. no. 05-665	1:1000
rabbit monoclonal anti-β-catenin	Cell Signalling Technology	cat. no. 8480S	1:1000
mouse monoclonal anti-β-actin	Abcam	ab8226	1:10 000
monoclonal anti-GAPDH, HRP-linked	Proteintech	HRP-60004	1:5000
goat anti-rabbit IgG, HRP-linked	Cell Signalling Technology	cat. no. 7074S	1:2500
goat anti-mouse IgG (H+L), HRP-linked	Thermo Fisher Scientific	cat. no. 31430	1:2500
rabbit anti-sheep IgG (H+L), HRP-linked	Thermo Fisher Scientific	cat. no. 31480	1:2500

### 2.11. Immunoprecipitation

For anti-GFP immunoprecipitations (IPs), anti-GFP beads (MRC PPU Reagents and Services) were pre-equilibrated using NP-40 lysis buffer. After an aliquot of the lysate was stored as the Input sample, 10 µl pre-equilibrated beads (50% slurry) was mixed with the clarified lysate (1 mg protein) and incubated on rotation for 2 h at 4°C. For anti-CK1α IPs, anti-CK1α antibody (MRC PPU Reagents and Services, 1 µg mg^−1^ protein in lysate) were first incubated with lysate (250 µg protein) on rotation (16 h, 4°C) before 20 µl protein G sepharose beads were added (1 h, 4°C) to pull-down anti-CK1α antibody and co-precipitating proteins. Beads were pelleted by centrifugation (1200 rpm, 5 min), post-IP supernatant was collected as flow-through, and beads washed 3 × 0.5 ml lysis buffer before adding 50 µl NuPAGE 1 × lithium dodecyl sulfate (LDS) sample buffer. Samples were eluted by boiling at 95°C for 10 min. Input, IP and flow-through samples were analysed by SDS-PAGE, transferred to PVDF membranes and western blotting.

### 2.12. Quantitative real-time PCR and primers

Cells were seeded onto 6-well plates at 70% confluency. Cells were washed once with 1 × PBS before incubation with either L-CM or Wnt3A-CM for 6 h. RNA was extracted from the cells using the RNeasy Mini Kit (74104, QIAGEN) and RNA concentration was quantified using NanoDrop (Thermo Fisher Scientific). cDNA synthesis was performed using 1 µg RNA with the iScript cDNA synthesis kit (Bio-rad). Each well of a 384-well quantitative real-time PCR (qRT-PCR) plate contained the following reaction mixture (10 µl): 2 µl cDNA (diluted 1 : 10), 50% (v/v) iQ-SYBR Green supermix (Bio-Rad), 2 µM forward primer and 2 µM reverse primer. Reactions were performed on a CFX384 machine (Bio-Rad). Primers: *Axin2:* forward-TACACTCCTTATTGGGCGATCA, reverse-TTGGCTACTCGTAAAGTTTTGGT; *GAPDH:* forward-TGCACCACCAACTGCTTAGC, reverse-GGCATGGACTGTGGTCATGAG. Fold change was determined using the comparative Ct method (ΔΔCt) for Wnt target gene *Axin2* expression normalized to the expression of housekeeping gene *GAPDH*. Microsoft Excel software was used for data processing and GraphPad Prism 9 was employed for statistical analysis and graph preparation. At least three biological replicates and three technical replicates for each condition were employed.

## 3. Results

### 3.1. A novel homozygous variant identified in 60-year-old female patient diagnosed with PPK

**Figure 1.**
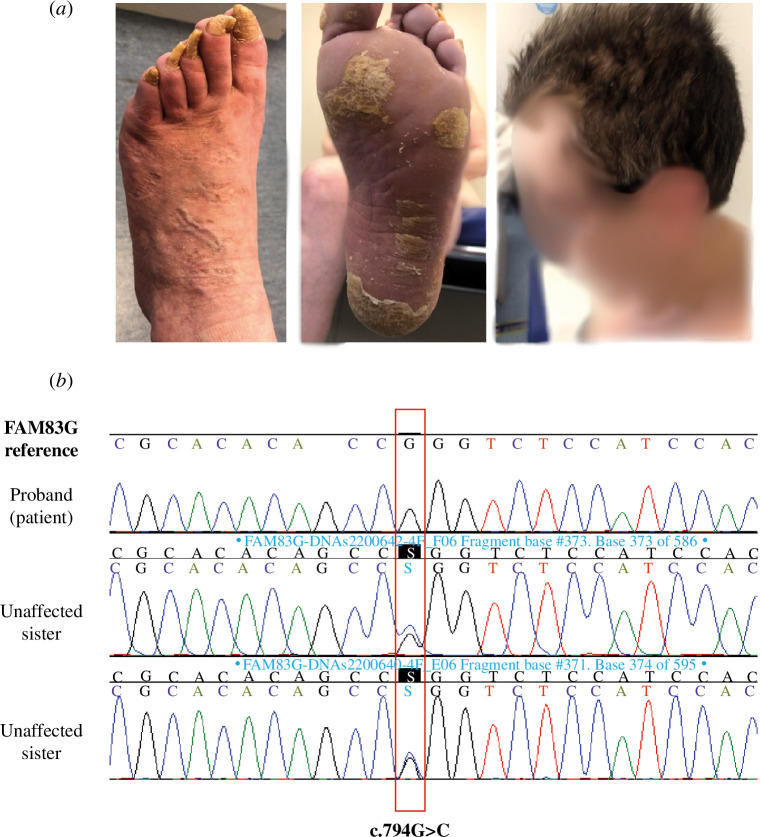
A novel homozygous *FAM83G* variant identified in a patient with PPK. (*a*) Clinical photographs taken from the patient show dystrophic nails (left panel), hyperkeratosis on the foot (middle panel), and thin, curly and sparse hair (right panel—patient’s face is blurred to obscure identifying features). (*b*) WES results from the patient displaying the genetic variant c.794G>C p.(Arg265Pro) within the *FAM83G* gene against a reference sequence and Sanger sequencing results in the unaffected siblings showing the heterozygous variant.

The 60-year-old female PPK patient reported a history of uneventful pregnancy, birth and normal early development. She reported that she first exhibited symptoms, such as alterations in the skin of both hands and feet, her hair, and dystrophic nails at the age 3. She was diagnosed with PPK at this stage. In the intervening years, physical examination revealed recurring features including thin, curly and sparse hair, long upper eyelid eyelashes, poor dental enamel and PPK, indications that persist presently (figure 1*a*). Her family history includes parental consanguinity and two unaffected siblings. Written informed consent, which was approved by the Vall d’Hebron University Hospital ethics committee, was obtained from the patient prior to obtaining and using clinical materials and photographs. Whole exome sequencing (WES) performed on peripheral blood DNA from the patient identified a homozygous *FAM83G* gene variant (c.794G>C, p.Arg265Pro; figure 1*b*). The homozygous variant identified using gnomAD version 4.0 was predicted to be uncertain according to REVEL (0.29) but pathogenic according to AlphaMissense (0.986). Sanger sequencing performed on two unaffected siblings were both heterozygous, with c.794G>C variant seen only on one of the alleles (figure 1*b*). These results suggested an autosomal recessive trait for the phenotypic manifestation.

### 3.2. FAM83G^R265P^ variant displays reduced stability, inability to interact with CK1α and reduced WNT activity

**Figure 2.**
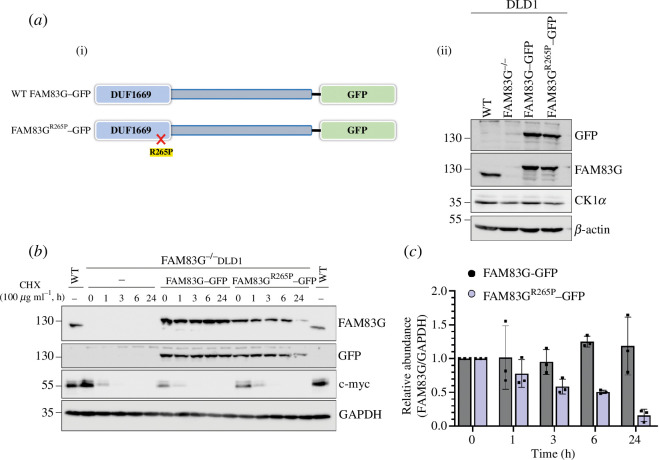
FAM83G^R265P^-GFP stably expressed in FAM83G^−/−^ DLD1 cells has lower stability. (*a*) WT DLD1 cells or FAM83G^−/−^ DLD1 cells or those transduced with retroviruses to stably express either FAM83G-GFP or FAM83G^R265P^-GFP were lysed and extracts (20 µg protein) resolved by SDS-PAGE before transferring onto PVDF membranes. The abundance of FAM83G-GFP and FAM83G^R265P^–GFP was confirmed using immunoblotting with anti-GFP and anti-FAM83G antibodies. β-actin was used as a loading control. (*b*) FAM83G^−/−^ DLD1 cells or those expressing FAM83G-GFP and FAM83G^R265P^–GFP were treated with cycloheximide (100 µg ml^−1^) and lysed at the indicated time points after treatment. Extracts (20 µg protein) were resolved by SDS-PAGE before transferring onto PVDF membranes, which were subjected to immunoblotting with the indicated antibodies. (*c*) Western blot quantification of signals from (*b*) where FAM83G signal was quantified using Fiji 1.53q (ImageJ), normalized to GAPDH signal, and presented relative to 0 h CHX treatment control (*n* = 3, error bars represent mean ± s.d.).

**Figure 3.**
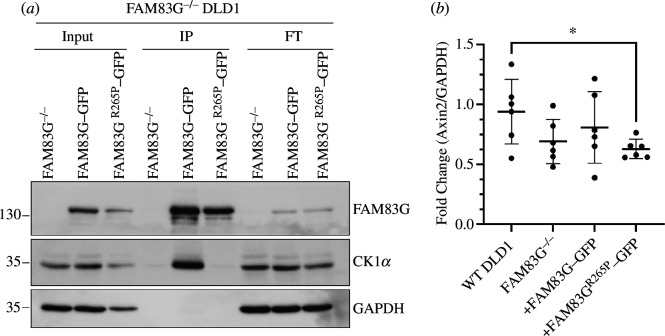
FAM83G^R265P^-GFP does not interact with CK1α and fails to activate the canonical WNT signalling pathway. (*a*) Extracts from FAM83G^−/−^ DLD1 cells stably expressing WT FAM83G-GFP and FAM83G^R265P^-GFP were subjected to anti-GFP IPs. Input extracts (20 µg protein), IPs and post-IP flow-through extracts (20 µg protein) were resolved by SDS-PAGE, transferred to PVDF membranes, and analysed by immunoblotting using the indicated antibodies. (*b*) WT DLD1 cells or cells from (*a*) were lysed, followed by RNA isolation and cDNA synthesis. qRT-PCR was performed using the cDNA to measure *Axin2* and *GAPDH* transcript levels. *Axin2* mRNA levels were normalized to *GAPDH* expression for all samples. Individual data points are shown on a scatter graph with an overlay of the mean ± s.d. Statistical analysis was performed using an unpaired Student’s *t*‐test with Welch’s correction, *p < 0.05 (*n* = 6).

A GFP-tag was introduced at the C-terminus of WT FAM83G and the FAM83G^R265P^ mutant and both were expressed in FAM83G^−/−^ DLD1 cells [19] using retroviral transductions (figure 2*a*). Analysis of protein expression by immunoblotting revealed slightly lower expression of FAM83G^R265P^-GFP in comparison to WT FAM83G-GFP (figure 2*a*). To assess whether this could be due to the reduced stability of the FAM83G^R265P^ mutant, cells were subjected to cycloheximide (CHX) treatment to inhibit new protein synthesis and protein abundance was monitored at specific time points up to 24 h by immunoblotting. Compared with WT FAM83G-GFP, which appeared stable over 24 h following CHX treatment, FAM83G^R265P^–GFP levels were dramatically reduced to about 50% by 6 h and 70% by 24 h following CHX treatment (figure 2*b,c*)*,* suggesting reduced stability of the FAM83G^R265P^-GFP. As expected, c-myc levels reached almost undetectable levels within 1 h of CHX treatment and remained undetectable thereafter (figure 2*b*). FAM83G exists in a stable complex with CK1α in cells [12,16]. Therefore, whether the pathogenic FAM83G^R265P^ mutant could interact with CK1α was assessed. While anti-GFP immunoprecipitates (IPs) of WT FAM83G-GFP from cell extracts robustly co-precipitated endogenous CK1α, the FAM83G^R265P^-GFP IPs did not (figure 3*a*), suggesting the inability of the FAM83G^R265P^ mutant to bind to CK1α.

FAM83G is reported to activate canonical WNT signalling via its interaction with CK1α [16]. Consistent with this, the levels of *Axin2* transcripts observed in WT DLD1 cells, which display enhanced WNT activity owing to an inherent *APC* mutation [20], were attenuated in FAM83G^−/−^ DLD1 cells (figure 3*b*). Restoration of WT FAM83G-GFP in FAM83G^−/−^ DLD1 cells partially restored *Axin2* mRNA expression but the FAM83G^R265P^-GFP mutant did not (figure 3*b*), indicating that the mutant failed to induce WNT activity.

### 3.3. FAM83G^R265P^ protein instability and its inability to interact with CK1α persists in patient skin fibroblasts

**Figure 4.**
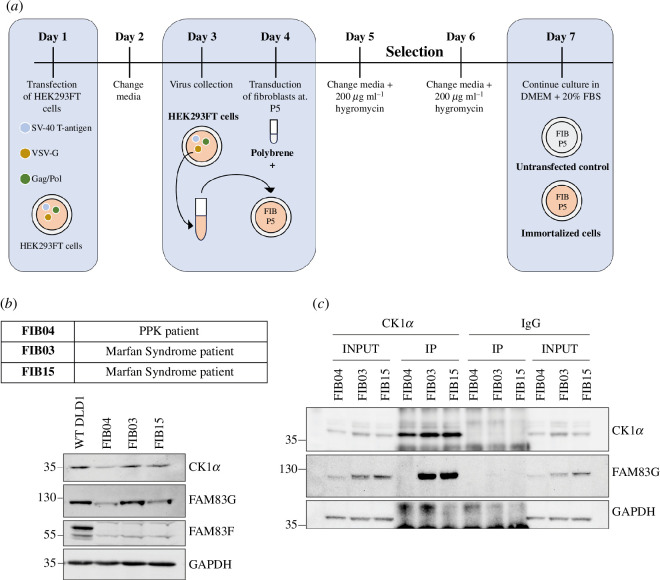
Reduced abundance of FAM83G and CK1α levels and loss of FAM83G–CK1α interaction in skin fibroblasts derived from PPK patient. (*a*) Schematic outline of the protocol used for immortalization of human skin fibroblast cells using retroviral transduction and SV−40 T-antigen overexpression. Retroviruses encoding SV−40 T-antigen were produced in HEK293FT cells and employed to transduce cultured skin fibroblast cells at passage 5. Transduced cells were selected in hygromycin and surviving immortalized fibroblast cells were taken forward. (*b*) WT DLD1 cells, control skin fibroblasts derived from Marfan syndrome patients (FIB15, FIB03) and PPK patient skin fibroblasts (FIB04) were lysed and extracts (20 µg protein) resolved by SDS-PAGE and transferred to PVDF membranes, which were subjected to immunoblotting using the indicated antibodies. (*c*) Clarified cell extracts (1 mg total protein) for each indicated skin fibroblast cell line were incubated with either CK1α antibody or IgG control (1 µg mg^−1^ extract protein) for 16 h on a rotating platform. Protein G sepharose beads were added to isolate co-precipitating proteins. Input extracts and IPs were subjected to SDS-PAGE and transferred to PVDF membranes, which were analysed by immunoblotting using the indicated antibodies.

Fibroblasts from skin biopsies of the PPK patient harbouring the homozygous *FAM83G* c.794G>C (protein FAM83G^R265P^) variant (FIB04) were collected along with two age- and sex-matched controls (FIB03 and FIB15). Written informed consent, which was approved by the Vall d’Hebron University Hospital ethics committee, was obtained from the patients for the generation and use of skin fibroblasts prior to skin biopsies. For biochemical assays, all skin fibroblast types were first immortalized by introducing SV-40 T-antigen through retroviral transductions (figure 4*a*). In both FIB03 and FIB15 control fibroblast cells, FAM83G protein levels were comparable to those observed in WT DLD1 cells, but in FIB04 skin fibroblasts from the PPK patient FAM83G protein levels were substantially lower (figure 4*b*; electronic supplementary material, figure S1), indicating that the R265P mutation affects FAM83G protein stability as observed above. All fibroblast cells displayed lower levels of CK1α compared with WT DLD1 cells, however, FIB04 cells displayed even lower CK1α levels than FIB03 and FIB15 controls (figure 4*b*; electronic supplementary material, figure S1). FAM83F protein, which also activates WNT signalling through an interaction with CK1α [15], was not detected in any of the skin fibroblast cells (figure 4*b*). Next, FAM83G-CK1α interaction at the endogenous level in skin fibroblasts was assessed. FAM83G was detected in anti-CK1α IPs from control FIB03 and FIB15 fibroblast cell extracts but not from PPK FIB04 extracts (figure 4*c*). No FAM83G was detected in control anti-IgG IPs from any cell line and, as expected, only anti-CK1α IPs enriched CK1α from all three cell lines (figure 4*c*). These data confirm that the pathogenic FAM83G^R265P^ variant renders it incapable of interacting with CK1α at the endogenous level.

### 3.4. Reduced WNT signalling response in skin fibroblast cells from the PPK patient

**Figure 5.**
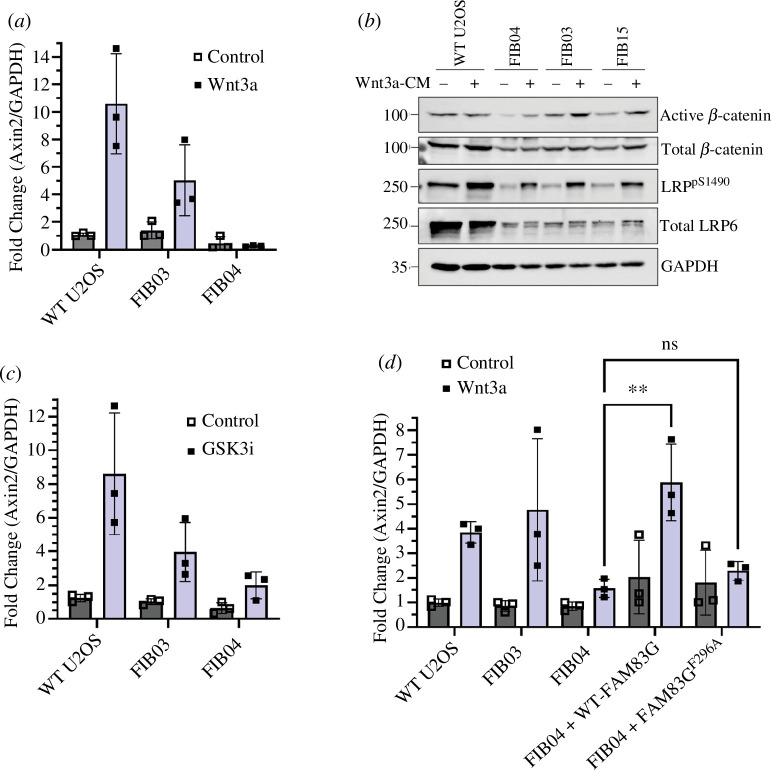
Reduced WNT signalling activity in skin fibroblasts derived from the PPK patient. (*a*) WT U2OS, control fibroblasts (FIB03) and PPK patient skin fibroblasts (FIB04) were incubated with L-CM or Wnt3A-CM for 6 h before RNA extraction, cDNA synthesis and analysis of *Axin2* and *GAPDH* transcripts by qRT-PCR. *Axin2* mRNA expression was normalized to *GAPDH* and the mean of three biological replicates was plotted as a bar graph ± s.d. (*b*) WT U2OS, control fibroblasts (FIB15, FIB03) and patient skin fibroblasts (FIB04) were incubated with L-CM and Wnt3A-CM for 6 h before lysis. Extracts (20 µg protein) were resolved by SDS-PAGE and transferred to PVDF membranes, which were analysed by immunoblotting using the indicated antibodies. (*c*) As in (*a*), except the cells were pre-treated with DMSO or the GSK3 inhibitor CHIR99021 (GSK3i) at 5 µM for 6 h before RNA extraction, cDNA synthesis and qRT-PCR. (*d*) WT U2OS, control fibroblasts (FIB03), patient skin fibroblasts (FIB04) and two PPK patient cell lines retrovirally transduced to overexpress either WT FAM83G or CK1-binding deficient mutant FAM83G^F296A^ were incubated with L-CM or Wnt3A-CM for 6 h and processed as in (*a*) for qRT-PCR to measure normalized *Axin2* mRNA expression. Statistical analysis was performed using two-way ANOVA with multiple comparisons, **p < 0.01, ns = no statistical significance (*n* = 3, error bars represent ± s.d.).

Next, the effect on WNT signalling activity in PPK fibroblasts by FAM83G^R265P^ expression was interrogated. U2OS osteosarcoma, FIB03 and FIB04 cells were incubated in the presence of control or Wnt3A conditioned medium for 6 h, and the expression of WNT target gene *Axin2* was measured by qRT-PCR. In both U2OS and FIB03 control cells, Wnt3A treatment induced a significant increase in *Axin2* transcript levels (figure 5*a*). In contrast, in PPK patient FIB04 skin fibroblasts, Wnt3A did not induce any increase in *Axin2* transcript levels compared with controls (figure 5*a*), suggesting that either the cells do not respond to Wnt3A stimulation or the transcription of WNT genes is impaired via disruption of WNT signalling. In all cells, including PPK patient-derived FIB04 cells, increased levels of active β-catenin [21] and LRP6^S1490^ phosphorylation [22] were evident upon Wnt3A treatment compared with controls (figure 5*b*; electronic supplementary material, figure S2), suggesting that WNT signalling activation by Wnt3A was intact. As a secondary method to investigate the WNT signalling response, the cells were incubated with an inhibitor of GSK3β. In the absence of a WNT ligand, β-catenin is continually degraded through the sequential phosphorylation of β-catenin by CK1α and GSK3β [23,24]. Therefore, inhibition of GSK3β activates WNT signalling by stabilizing β-catenin. In WT U2OS and control FIB03 skin fibroblasts, a significant increase in *Axin2* transcript expression compared with control was observed after treatment with GSK3β inhibitor (figure 5*c*). In contrast, in FIB04 fibroblasts, the GSK3β inhibitor elicited only a slight increase in *Axin2* transcript levels (figure 5*c*), further suggesting defective WNT signalling activity. Finally, whether restoring WT FAM83G in PPK patient-derived FIB04 fibroblasts could rescue WNT signalling was investigated. Indeed, compared with untransduced FIB04 skin fibroblasts, in FIB04 cells stably expressing WT FAM83G, Wnt3A induced a substantial increase in *Axin2* mRNA levels (figure 5*d*), indicating that the presence of the FAM83G^R265P^ mutation in FIB04 cells causes defective WNT signalling responses. Furthermore, FIB04 cells stably expressing FAM83G^F296A^, a well-established CK1α-binding deficient mutant [12], did not significantly rescue *Axin2* levels to the same extent as the WT protein, demonstrating that restoration of WNT signalling activity was dependent on FAM83G-CK1α interaction.

## 4. Discussion

Here, a novel homozygous variant c.794G>C in the *FAM83G* gene of a PPK patient is described which encodes the expression of FAM83G^R265P^ mutant protein. The patient displayed skin and hair phenotypes that were similar to those previously reported in two siblings harbouring the homozygous *FAM83G^A34E^* variant [9] and in dogs harbouring the homozygous *FAM83G^R52P^* variant [10]. Further characterization of the FAM83G^R265P^ mutant protein revealed that it displayed reduced stability, and an inability to interact with CK1α or activate the canonical WNT signalling pathway, similar to what was observed previously with FAM83G^A34E^ and FAM83G^R52P^ mutant proteins that also cause PPK in humans and dogs, respectively [18]. Our findings strongly imply that the loss of CK1α binding and the subsequent attenuation of WNT signalling underlie the pathogenesis of PPK caused by *FAM83G* variants.

Loss of FAM83G-CK1α interaction and defective WNT signalling at the endogenous level in skin fibroblasts derived from the PPK patient have been shown. The siblings of the patient, who displayed no symptoms of PPK, were heterozygous for the *FAM83G* c.794G>C variant, suggesting autosomal recessive transmission for disease pathology. Interestingly, restoration of the WT FAM83G protein in skin fibroblast cells derived from the PPK patient, which was intended to mimic a heterozygous trait displayed by unaffected siblings of the patient, was able to rescue defective WNT signalling evident in the patient cells. The precise molecular mechanism by which FAM83G-CK1α complexes regulate the WNT pathway, and maintain homeostasis of the cells, that eventually lead to the defects prevalent in PPK patients remain unresolved. Nonetheless, restoring the defective FAM83G-CK1α interaction and WNT signalling in affected areas of PPK patients could be a way to intervene against PPK pathology. It would also be interesting to investigate whether correction of the c.794G>C variant back to WT in skin fibroblast cells derived from the PPK patient by CRISPR/Cas9 genome editing could restore FAM83G stability, FAM83G-CK1α interaction and defective WNT signalling in these cells.

It is possible that the prognosis of PPK stems from dysregulated signalling between the epidermal, dermal and hypodermal layers of the skin leading to mistimed cell differentiation and proliferation [25]. Tight spatiotemporal control along the regulatory axis of several pathways including WNT, BMP and Shh signalling is important to maintain the healthy regeneration and multi-layered structure of the skin [26]. It is conceivable that FAM83G when mutated may lead to dysregulated WNT signalling within each layer of the skin, triggering improper skin development and an increased number of terminally differentiated cells on the outer epidermis which becomes thickened skin in PPK patients. Analysis of other WNT target genes in addition to Axin2, such as E-cadherin and fibronectin [27,28], in patient fibroblasts as well as profiling their physiological characteristics, may strengthen findings presented here and support a role for FAM83G^R265P^ in defective skin development and pathogenicity of PPK.

## Data Availability

Raw data associated with this manuscript is accessible at Mendeley Data Server at https://data.mendeley.com/datasets/jc2zxjysmr/1. Supplementary material is available online [29].
